# Long-term safety and efficacy of CART-20 cells in patients with refractory or relapsed B-cell non-Hodgkin lymphoma: 5-years follow-up results of the phase I and IIa trials

**DOI:** 10.1038/sigtrans.2017.54

**Published:** 2017-10-09

**Authors:** Wen-ying Zhang, Yang Liu, Yao Wang, Chun-meng Wang, Qing-ming Yang, Hong-li Zhu, Wei-dong Han

**Affiliations:** 1 Biotherapeutic Department, Chinese PLA General Hospital, Beijing, China; 2 Department of Geriatric Hematology, Chinese PLA General Hospital, Beijing, China; 3 Department of Immunology, Institute of Basic Medicine, School of Life Sciences, Chinese PLA General Hospital, Beijing, China

For refractory or relapsed (r/r) B-cell non-Hodgkin lymphoma (NHL), the response rates to conventional salvage chemotherapy are 27–44%.^1^ Chimeric antigen receptors (CARs) efficiently redirect T-cell specificity and cytotoxicity to cells expressing the targeted Ag in an HLA-independent manner.^2^ The early phase clinical trials of CART cells for (r/r) B-cell NHL have demonstrated promising results. Recent updated data from US National Cancer Institute (NCI) showed that five of the seven (71%) evaluable patients with relapsed diffuse large B-cell lymphoma (DLBCL) obtained complete remissions (CRs) after infusion of anti-CD19 CAR-T cells, and four of the five CRs had long-term durability with duration ranging from 38 to 57 months.^3^ Encouraging results have also been seen in our prior studies of autologous CART-20 cells in patients with r/r CD20^+^ B-NHL (NCT01735604).^4,5^ This paper reports the long-term efficacy and safety of CART-20 cells in patients with r/r CD20^+^ B-NHL after 5-year follow-up.

From July 2012 to June 2015, a total of 17 patients with r/r B-cell NHL have been enrolled in our studies. As of July 2017, the median follow-up time was 20 months (range, 4–60 months). The patients underwent cytoreductive chemotherapy for tumor debulking and lymphodepletion between 3 and 7 days before T-cell infusion. All patients received at least one cycle CAR.20-CD137ζ transduced T cells infusion at a dose of 0.5–1.5×10^7^  kg^−1^. Clinical trial design and assay protocols have been reported in detail in our prior publications.^4,5^

The baseline characteristics of all the patients are presented in Table 1. Briefly, all patients were heavily pretreated and had received rituximab previously; 16 patients (94%) had received 4 or more previous treatment regimens, and 12 patients (70.6%) had relapsed after previous second-line chemotherapy regimens. One patient had relapsed post-autologous stem cell transplantation (SCT). Eleven patients (64.7%) were defined as either refractory or progressive according to their responses to recent chemotherapeutics. Fourteen patients (82.4%) were diagnosed with DLBCL and three (17.6%) had indolent lymphoma. Notably, five patients had bulky tumor burden in the phase I trial.

In phase IIa trial, 11 patients were available to evaluate the objective clinical responses. The overall objective response rate was 9 of 11 (81.8%), with 54.5% of patients (6/11) achieving CR and 27.3% (3/11) achieving partial remission (PR). One patient with PR and one patient with stable disease after CART-20 cell infusion received consolidative local radiotherapy and were subsequently converted to CR. In the phase I trial, five of six patients experienced tumor regression.

Twelve patients with remission but refusing autologous or allogeneic SCT were followed up for a median of 20 months (range, 4–60 months) from their first CART-20 cell infusion. The estimated median progression-free survival (PFS) of the 12 patients was 10 months (range, 2–57 months) (Figure 1a) and the estimated 2-year rate of PFS was 41.7% (5/12). The longest duration of response (57 months) was seen in UPNII-11 (Figure 1b). The UPNII-09 with advanced refractory marginal zone lymphoma had achieved remission of skin and bone marrow after the first CART-20 cell infusion, but still had enlarged spleen. Thus, UPNII-09 received the consolidated second infusion, and the size of the spleen became gradually smaller and continued to shrink for up to 36 months (Figure 2a). B-cell aplasia of this patient sustained for 150 weeks (Figure 2b and Supplementary Figure).

The treatment regimen was generally well tolerated. The acute adverse events included temporary chills and fever. The long-term monitoring for adverse events was for 5 years. The delayed adverse events related to CART-20 therapy were summarized in Table 2. No Grade 4 toxicities were observed. Grade 3 herpes zoster due to long-term hypogammaglobulinemia was observed in UPNII-09 at 7 months after infusion. The decrease of immunoglobulin occurred in all patients with B-cell lack. We preventatively administered intravenous immunoglobulin to avoid hypogammaglobulinemia until the B-cell recovery. During the long follow-up periods, no patient was found to have susceptibility to viral infection or increase of other diseases incidence.

Some similar studies of CART cell therapy of B-cell NHL have been reported since 2001.^6,7^ At the 2016 annual meeting of the American Society of Hematology, updated data from Transcend NHL 001 trial showed that the longest observable PFS of patients with DLBCL was 9 months in those who achieved CR after treatment with CART-19 cells.^8^ However, in our trials, the longest continued complete remission time of a patient with DLBCL was 57 months, which was comparable to the updated data from NCI group’s trial of CART-19 in DLBCL.^3^

In conclusion, long-duration CRs were observed in our CART-20 cell therapy. Compared to the outcomes of other clinical trials evaluating CAR-T cells in r/r B-NHL, our CART-20 cell therapy possibly made patients get longer PFS time. Our results provided unique evidence supporting the efficacy and safety of CART-20 in patients with r/r B-cell NHL.

## Figures and Tables

**Figure 1:**
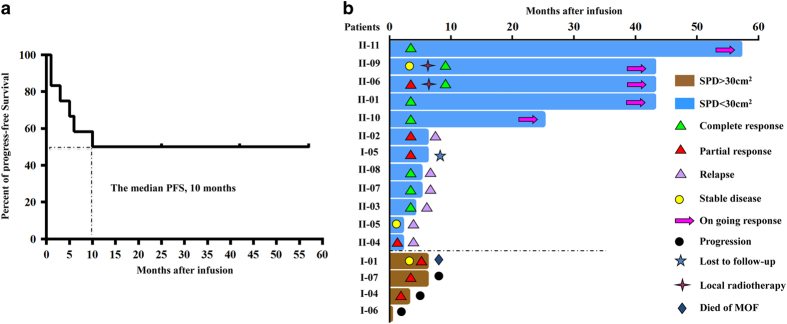
Kaplan–Meier curves for PFS and duration responses of all eligible patients. (**a**) The median PFS was 10 months (95% CI, 2–57 months). (**b**) Twelve patients with SPD<30 cm^2^ had clinical improvement; four patients with bulky tumors suffered lymphoma progression.

**Figure 2:**
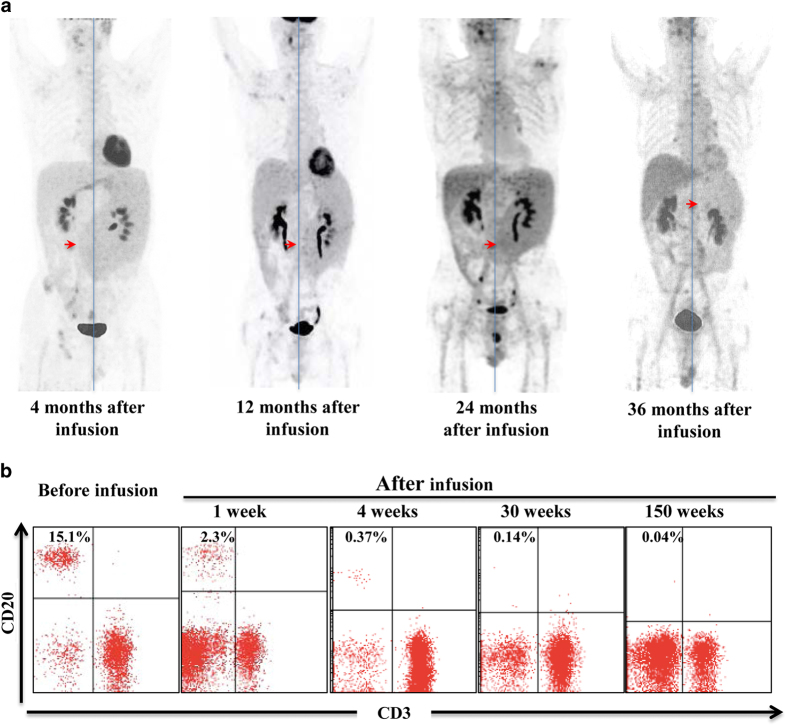
The change of the spleen size and CD20^+^ B-cell counts in the PB of patients UPNII-09. (**a**) The whole-body 2-[18F]-fluoro-2-deoxy-D-glucose positron emission tomography (FDG-PET) scans show the larger spleen over the midline of body at 4 months after infusion, then shrink to midline and left midline, respectively, at 12 and 24 months. Especially at 36 months after infusion, the size of spleen become almost normal. (**b**) The CD20^+^ lymphocyte cells determined by flow cytometry in PB after CART-20 cell infusion is shown. From 1 week after infusion, the proportion began to decline, and it lasted for over 150 weeks.

**Table 1:** Baseline demographic and clinical characteristics

*Characteristic*	*All patients (*n*=17, 100%)*
Median age, years (range)	57 (25–85)
	
*Sex, no. (%)*	
Female	3 (17.6%)
Male	14 (82.4%)
	
*Disease stage*	
I, II	4 (23.5%)
III, IV	13 (76.5%)
	
*IPI group*	
Intermediate: 2–3	15 (88.2%)
High: 4–5	2 (11.8%)
Ki-67: Median (range), %	75 (60–90%)
BM involvement	3 (17.6%)
Median (range) time from diagnosis, years	3 (0.25–37)
Median (range) time from last chemotherapy regimen, months	4 (1–6)
Median (range) number of previous treatment regimens	7 (2–14)
	
*Type of prior treatment regimen*	
One or more rituximab treatments	17
Radiotherapy	2
AHSCT	1
	
*Pathology, no. (%)*	
Indolent lymphoma	3 (17.6%)
Follicular lymphoma	1 (5.9%)
Mantle cell lymphoma	1 (5.9%)
Primary cutaneous marginal zone lymphoma	1 (5.9%)
Diffuse large B-cell lymphoma	14 (82.4%)
Relapsed after previous therapy with R-CHOP	1 (5.9%)
Relapsed after previous therapy with second-line chemotherapy	12 (70.6%)
Relapsed after previous AHSCT	1(5.9%)
	
*Tumor burden SPD*	
SPD>30	5 (29.4%)
SPD<30	12 (70.6%)
	
*Conditioning therapy*	
None	4 (23.5%)
FC	1 (5.9%)
Others	12 (70.6%)

Abbreviations: AHSCT, autologous hematopoietic stem cell transplantation; BM, bone marrow; CHOP, cyclophosphamide, doxorubicin, vincristine, prednisone; FC, fludarabine and cyclophosphamide; IPI, International Prognostic Index; SPD, the sum of the products of diameters of all index lesions.

Eastern Cooperative Oncology Group (ECOG) scores indicate the performance status of patients with respect to activities of daily living on a scale from 0 to 5, with higher numbers indicating greater disability.

**Table 2:** Adverse events

*Adverse events*	*No. of patients (%,* n*=16)*	
	*All grades*	*Grade ⩾3*
*Infusion-related events*		
Fever	16 (100.0%)	0
Rigors	15 (93.8%)	1 (6.2%)
Fatigue	3 (18.7%)	0
Hypotension	1 (6.2%)	0
Exudative inflammation of the lungs	2 (12.5%)	0
Cytokine release syndrome	4 (25.0%)	0
Acute alimentary tract hemorrhage	2 (12.5%)	1 (6.2%)
		
*Delayed-events after infusion*		
Hematologic events		
Neutropenia	3 (18.7%)	1 (6.2%)
Thrombocytopenia	3 (18.7%)	0
Lymphocytopenia	11 (68.8%)	0
Anemia	2 (12.5%)	0
Immunology events		
Serum immunoglobulin decrease	7 (43.8%)	1 (6.2%)
Allergic reaction/hypersensitivity	1 (6.2%)	0
Allergic rhinitis	1 (6.2%)	0
Autoimmune enteritis	1 (6.2%)	0
Vasculitis	1 (6.2%)	0
Herpes zoster	1 (6.2%)	1 (6.2%)
Viral hepatitis	0	0
Other virus infection	1 (6.2%)	0
Dermatomycoses	1 (6.2%)	0
Bacterial infection	1 (6.2%)	0
Nervous system disorder		
Numbness	1 (6.2%)	0
Insomnia	1 (6.2%)	0
Hypomnesis	1 (6.2%)	0
Laboratory abnormalities		
ALT elevation	2 (12.5%)	0
AST elevation	1 (6.2%)	0
Hyperuricaemia	2 (12.5%)	0
Hypoalbuminaemia	1 (6.2%)	0
Hypokalemia	1 (6.2%)	1
LDH elevation	2 (12.5%)	0

Abbreviations: ALT, alanine aminotransferase; AST, aspartate aminotrans- ferase; LDH, lactate dehydrogenase.
