# Supplementary material for: Long-term safety and efficacy of CART-20 cells in patients with refractory or relapsed B-cell non-Hodgkin lymphoma: 5-years follow-up results of the phase I and IIa trials
Source: Signal Transduct Target Ther. 2017 Oct 9;2:17054–. doi: 10.1038/sigtrans.2017.54 (PMC5661643; doi:10.1038/sigtrans.2017.54)
Supplement: Supplementary information [file sigtrans201754-s1.pdf]

Supplementary information

Long-term Safety and Efficacy of CART-20 Cells in Patients with Refractory or Relapsed B-Cell Non-Hodgkin Lymphoma: 5-years follow-up results of the phase I and IIa trials

Wen-ying Zhang [1*], Yang Liu [2*], Yao Wang[3*], Chun-meng Wang [1], Qing-ming Yang[1], Wei-dong Han [1,3#], Hong-li Zhu[2#]

1 Biotherapeutic Department, Chinese PLA General Hospital, Beijing 100853, China

2 Department of Geriatric Hematology, Chinese PLA General Hospital, Beijing 100853, China

3 Department of Immunology, Institute of Basic Medicine, School of Life Sciences, Chinese PLA General Hospital, Beijing 100853,China

*Contributed equally to this work

Supplement Figure

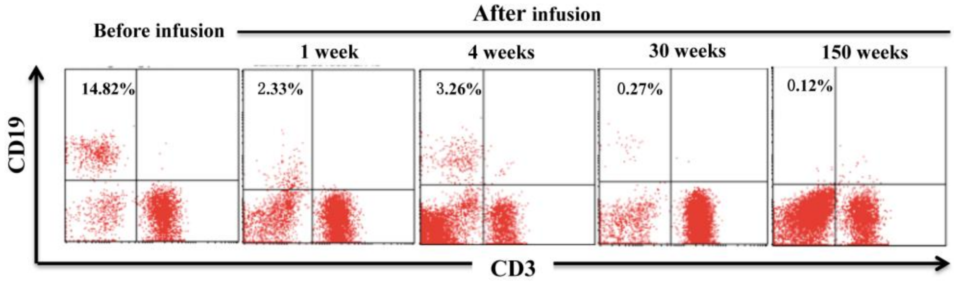

Supplement Figure. The $CD19^+$ lymphocyte cells also were determined by flow cytometry in PB after CART-20 cell infusion is shown. From 1 week after infusion, the proportion began to decline, and it lasted for over 150 weeks.
